# Historical and future trends in emergency pituitary referrals: a machine learning analysis

**DOI:** 10.1007/s11102-022-01269-1

**Published:** 2022-09-09

**Authors:** A. S. Pandit, D. Z. Khan, J. G. Hanrahan, N. L. Dorward, S. E. Baldeweg, P. Nachev, H. J. Marcus

**Affiliations:** 1 High-Dimensional Neurology, Queen Square Institute of Neurology, University College London, London, UK; 2 Victor Horsley Department of Neurosurgery, National Hospital for Neurology and Neurosurgery, London, UK; 3 Wellcome/EPSRC Centre for Interventional and Surgical Sciences, University College London, London, UK; 4 Department of Diabetes and Endocrinology, University College London Hospital, London, UK; 5 Centre for Obesity & Metabolism, Department of Experimental & Translational Medicine, Division of Medicine, University College London, London, UK

**Keywords:** Machine learning, Time-series forecasting, Referrals, Service demand, COVID-19

## Abstract

**Purpose:**

Acute pituitary referrals to neurosurgical services frequently necessitate emergency care. Yet, a detailed characterisation of pituitary emergency referral patterns, including how they may change prospectively is lacking. This study aims to evaluate historical and current pituitary referral patterns and utilise state-of-the-art machine learning tools to predict future service use.

**Methods:**

A data-driven analysis was performed using all available electronic neurosurgical referrals (2014–2021) to the busiest U.K. pituitary centre. Pituitary referrals were characterised and volumes were predicted using an auto-regressive moving average model with a preceding seasonal and trend decomposition using Loess step (STL-ARIMA), compared against a Convolutional Neural Network-Long Short-Term Memory (CNN-LSTM) algorithm, Prophet and two standard baseline forecasting models. Median absolute, and median percentage error scoring metrics with cross-validation were employed to evaluate algorithm performance.

**Results:**

462 of 36,224 emergency referrals were included (referring centres = 48; mean patient age = 56.7 years, female:male = 0.49:0.51). Emergency medicine and endocrinology accounted for the majority of referrals (67%). The most common presentations were headache (47%) and visual field deficits (32%). Lesions mainly comprised tumours or haemorrhage (85%) and involved the pituitary gland or fossa (70%). The STL-ARIMA pipeline outperformed CNN-LSTM, Prophet and baseline algorithms across scoring metrics, with standard accuracy being achieved for yearly predictions. Referral volumes significantly increased from the start of data collection with future projected increases (p < 0.001) and did not significantly reduce during the COVID-19 pandemic.

**Conclusion:**

This work is the first to employ large-scale data and machine learning to describe and predict acute pituitary referral volumes, estimate future service demands, explore the impact of system stressors (e.g. COVID pandemic), and highlight areas for service improvement.

**Supplementary Information:**

The online version contains supplementary material available at 10.1007/s11102-022-01269-1.

## Introduction

Pituitary tumours are among the most common intracranial tumours, presenting either incidentally, as a result of local mass effect or through symptoms following systemic endocrine dysfunction [1]. The diagnostic and management pathways for patients with these tumours are complex, involving numerous disciplines across primary and secondary care [2, 3]. Diverse clinical presentations, as well as multiple potential venues for interaction with healthcare services, can result in a wide range of patient pathways and journeys [2–4]. This poses a challenge to healthcare services and may contribute toward delays in correct diagnosis and definitive management (e.g., surgery) [5, 6]. This is of particular importance for patients requiring urgent treatment, including those presenting with rapidly declining vision and other acute neurological deficits.

To meet this challenge, there has been a transition toward a “centres of excellence” model of pituitary tumour care, whereby multidisciplinary neurosurgical and medical teams are integrated into a unified service, and patient pathways are consolidated within a specialist centre. While this expedites access to appropriate surgical and medical expertise, it also expands the catchment size and the complexity of referral and management networks for each specialist centre [2, 3].

Continuous data-driven auditing of these specialist networks is critical to understanding present pathways, potential future service demands and opportunities for improvement [2, 7]. This is particularly pertinent in the current healthcare landscape, where periodic stressors (i.e. COVID-19 pandemic waves) have significantly impacted service delivery across surgical disciplines, including pituitary surgery [8]. Manually performing such analyses, on the other hand, is time- and resource-intensive. Electronic databases, bespoke programming and machine learning offer an avenue for streamlined, automatic and accurate analysis of patient pathway data and prediction of future trends [9].

In this study, we sought to apply these technologies to analyse urgent pituitary and anterior skull base referrals made to the United Kingdom’s largest pituitary centre. We aimed to describe referral characteristics, network trends (including the impact of the COVID-19 pandemic), and highlight areas for improvement. As a subsidiary analysis, we evaluate and propose a time-series analysis framework, which can accurately predict future pituitary referrals and optimise future service delivery.

## Methods

### Reporting guidelines

In the absence of a dedicated checklist for time-series forecasting, the study was conducted in accordance with TRIPOD guidelines for predictive model development where relevant [10].

### Ethics and regulations

Our retrospective study and use of anonymised referral data was approved by the institutional review board (National Hospital for Neurology and Neurosurgery, London, UK) as a service evaluation (121-202021-CA) with the requirement for informed consent being waived.

### Data collection

Data processing and analysis were performed in Python 3.8.6, using a MacBook Pro (2017, 2.9 GHz, 16 GB RAM) using numpy (v = 1.19.5) and pandas (v = 1.2.3) libraries. Raw referral data from the centre’s cloud-based referral platform (referapatient.org) was securely obtained and extracted in comma-separated values format and downloaded to a hospital workstation before fully de-identifying the data and transferring it to the system aforementioned. Referrals were made via the electronic referral platform to the neurosurgical centre from June 2014 to October 2021. Included in this analysis were only urgent or emergency referrals, which had confirmed a lesion that originated from or infiltrated into the pituitary, infundibulum, hypothalamus, sella, suprasellar space, sphenoid bone or sinus and clivus. Also included were patients who were referred because of a suspected complication related to a recent pituitary, endonasal or anterior skull base procedure, and those who presented with a history suspicious for cerebrospinal fluid (CSF) rhinorrhoea. Excluded were patients with imaging findings demonstrating a lesion which did not involve the aforementioned areas, or a historical pituitary lesion unrelated to the referral that. Also excluded were patients with imaging findings exclusively of ‘empty sella’.

### Data analysis and visualisation

#### Time-series analysis

All time-series modelling was based on simple referral volume data. In preparation for this analysis, the referral volumes were first sorted into monthly brackets, rather than taking daily volumes, and used as the algorithm variable. This level of discretisation was chosen to account for observed short-term volatility and to increase the level of stationarity needed for accurate time-series modelling [11]. The choice of forecasting algorithm was based on evidence of clinical application of forecasting models with referral data using previously described hyperparameter tuning [9]. In brief, this included three algorithms: an automated pipeline which combined Seasonal and Trend decomposition using Loess (STL) with an automated regression integrated moving average (Auto-ARIMA) model, a Convolutional Neural Network-Long Short-Term Memory (CNN-LSTM) network [12, 13] and Prophet [14]. The implementation of each is now considered in turn.

ARIMA models are frequently used as a reference in domains such as econometrics [11]. Two changes were made in this case to enable automatic hyperparameter tuning and to make the model more resistant to time-series with an undetermined length, frame, and degree of seasonality. The raw data was first decomposed into seasonal, trend, and residual components using a Seasonal and Trend decomposition using Loess (STL). Each component was run through an automated grid search to identify the *p*, *d*, and *q* parameters, which specify the lag order, degree of differencing, and moving average order, respectively. The Akaike Information Criterion (AIC) was used to assess parameter value combinations in order to select the best set. If the seasonal and trend decomposition failed to enforce stationarity in the trend data, the auto-ARIMA step could model the trend, seasonality and residual separately before recomposing the data to forecast.

Deep learning approaches can automatically uncover and model hidden complexity within data and extract features of interest. CNNs can learn discriminative features by applying a non-linear transformation to time-series data, whereas LSTM networks use gating strategies to prevent short-term memory loss and increase learning and information processing inside the network. We divided the time series into sub-sequences with 12 “steps” (i.e., 1 year) as input and one output. This is then divided into two sub-samples, each with two targets, before being passed to the convolutional layer, which transforms the sub-samples before downsampling, flattening, and passing to a single LSTM layer with 64 neurons. To avoid overfitting, the dropout proportion was adjusted to 30%. The projected value was utilised to incrementally improve the training. Hyperparameter tuning to determine the number of convolutional filters and neurons was performed via grid search a priori.

Prophet is a Facebook-provided open-source library (https://facebook.github.io/prophet/). Here, the time series is split into four components: growth, yearly and weekly seasonality, and holidays, and then an additive regression model is fitted. Growth is represented as a piecewise linear or logistic growth trend, yearly seasonality is represented by a Fourier series, and weekly seasonality is represented by dummy variables. Prophet automatically recognises ‘changepoints’ in the trend during modelling, except for holidays or custom periods, which are entered by the user. In this case, the period of the Covid-19 lockdown in London, U.K. was manually specified as a custom period.

#### Evaluation of forecasting algorithm performance

The forecasting model performance was evaluated using error metrics, assessing performance for one-month, three-month, six-month and one-year periods. This range of time frames permits the evaluation of both short-term and long-term prediction abilities. Blocked cross-validation utilised all available data (June 2014 to October 2021), respecting the temporal order of the data, randomly divided into fivefolds with approximately 15-month prior time-frames for algorithm training. Median absolute error (MAE) and median absolute percentage error (MPE) were used as scoring metrics given the non-parametric distribution of the error metrics and frequent outliers. This process was repeated 1000 times, and the median average for each metric was calculated based on the out-of-sample component, with standard deviations estimated for each algorithm derived from the cross-validation. Two baseline models were used to compare the predictive algorithms used here, in line with standard recommendations [15].

#### Statistical analysis and data visualisation

Statistical comparisons of monthly referral volumes were implemented through scipy (v = 1.6.2). Tests of normality were performed using the Kolmogorov–Smirnov test, and homodescacity was checked using Bartlett’s test, before applying appropriate parametric or non-parametric tests. Post-hoc multiple comparisons were corrected for, by using the Benjamini–Hochberg method. Figures were produced using the *plotly* (v = 5.3.1) libraries within Python. To describe overall changes in referral patterns related to Covid-19, yearly time periods were compared pre-, during, and post-Covid-19 according to the dates set for U.K. government lockdown restrictions. A 12-month forecasted, out-of-sample period was also compared against, in order to test the time-series forecasting algorithm.

## Results

### Referral demographics

**Fig. 1:**
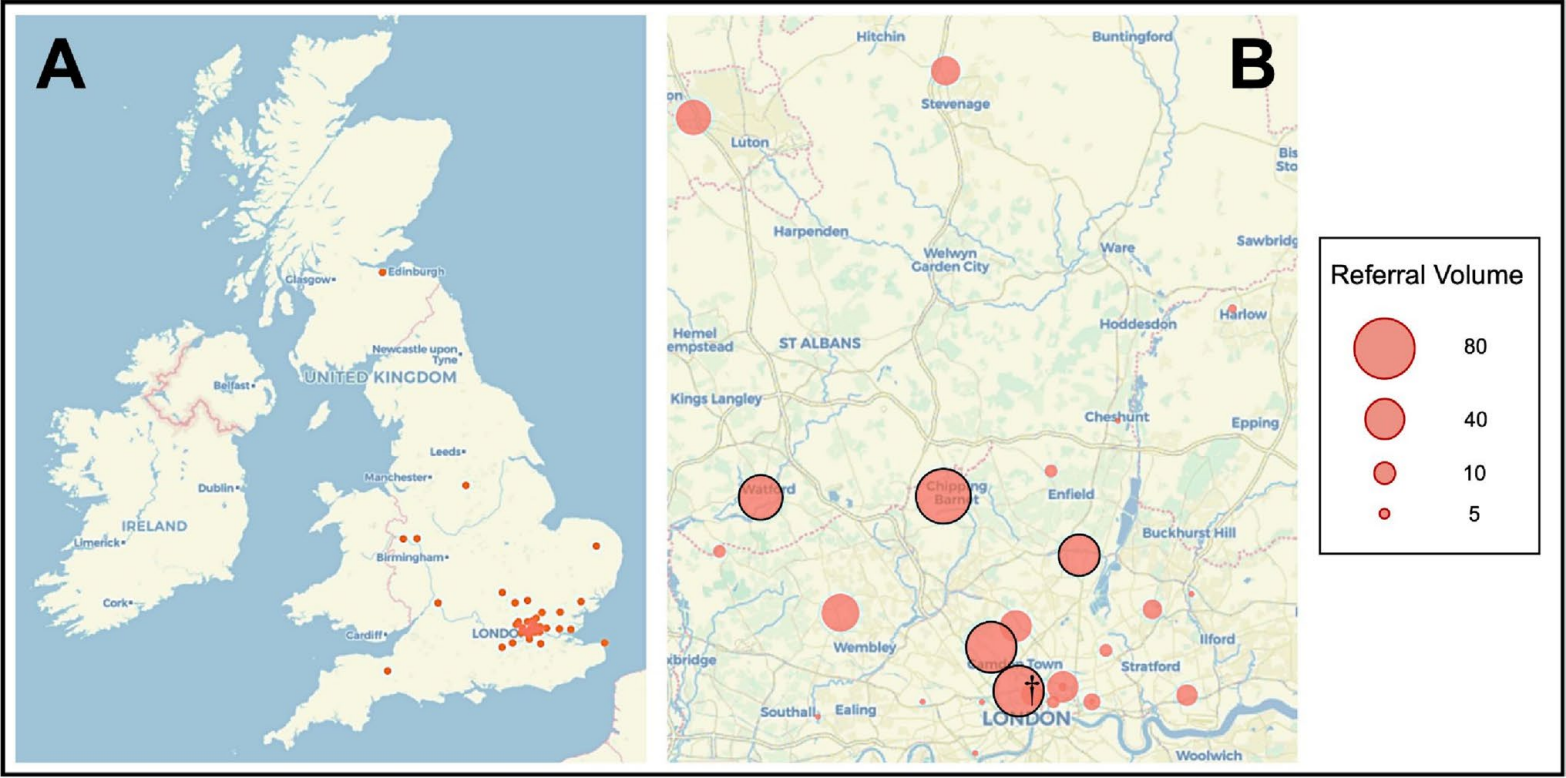
Geographical distribution of referrals. **A** Map of referring hospital sites (red dots) to the pituitary neurosurgical centre from across the U.K. **B** Northern Greater London referral catchment area with referring sites (red circles) size proportional to referral volume. The five highest volume referring sites (50 referrals or more) are highlighted with black borders. † denotes the approximate location of the receiving pituitary centre. Map underlay was obtained and adapted from openstreetmap.org under a Creative Commons licence (CC BY-SA 2.0)

Of the 36,224 emergency referrals made, 462 were eligible for inclusion—originating from 48 referring hospitals across England, Scotland and Wales (Table 1; Fig. 1A). 5 centres across the northern Greater London catchment area contributed to 54% of the total referrals (Fig. 1B).

**Table 1:** Referral demographics

Patients	462
Total number of referring centres	48
Mean age (years) [SD]	56.7 [20.2]
Gender (F/M [%])	228/234 [49.4/50.6]
Median Glasgow Coma Score [range, IQR]	15 [3–15]
Patients with a prior history of pituitary or anterior skull base surgery (%)	73 (15.8%)

### Geographical distribution of referrals

#### Breakdown by referring speciality, urgency and referrer

**Fig. 2:**
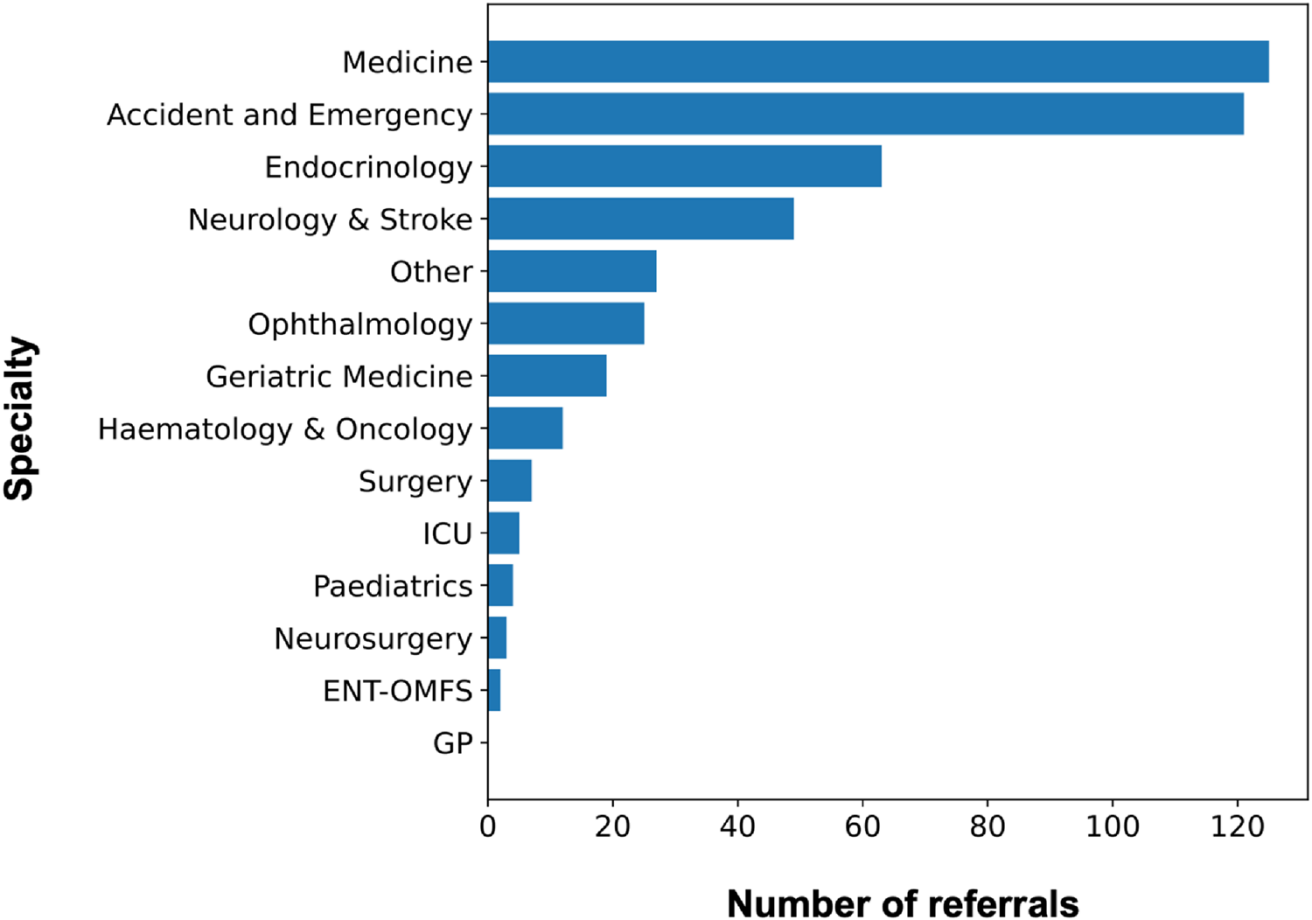
Total volume of referrals by individual specialities during the 7-year referral period. ‘Medicine’ includes cardiology, gastroenterology, respiratory, and other acute medical specialities. (*Haem* haematology, *Onc* oncology; *ICU* intensive care unit; *ENT* ear nose throat; *OMFS* oral and maxillofacial; *GP* general practice)

**Fig. 3:**
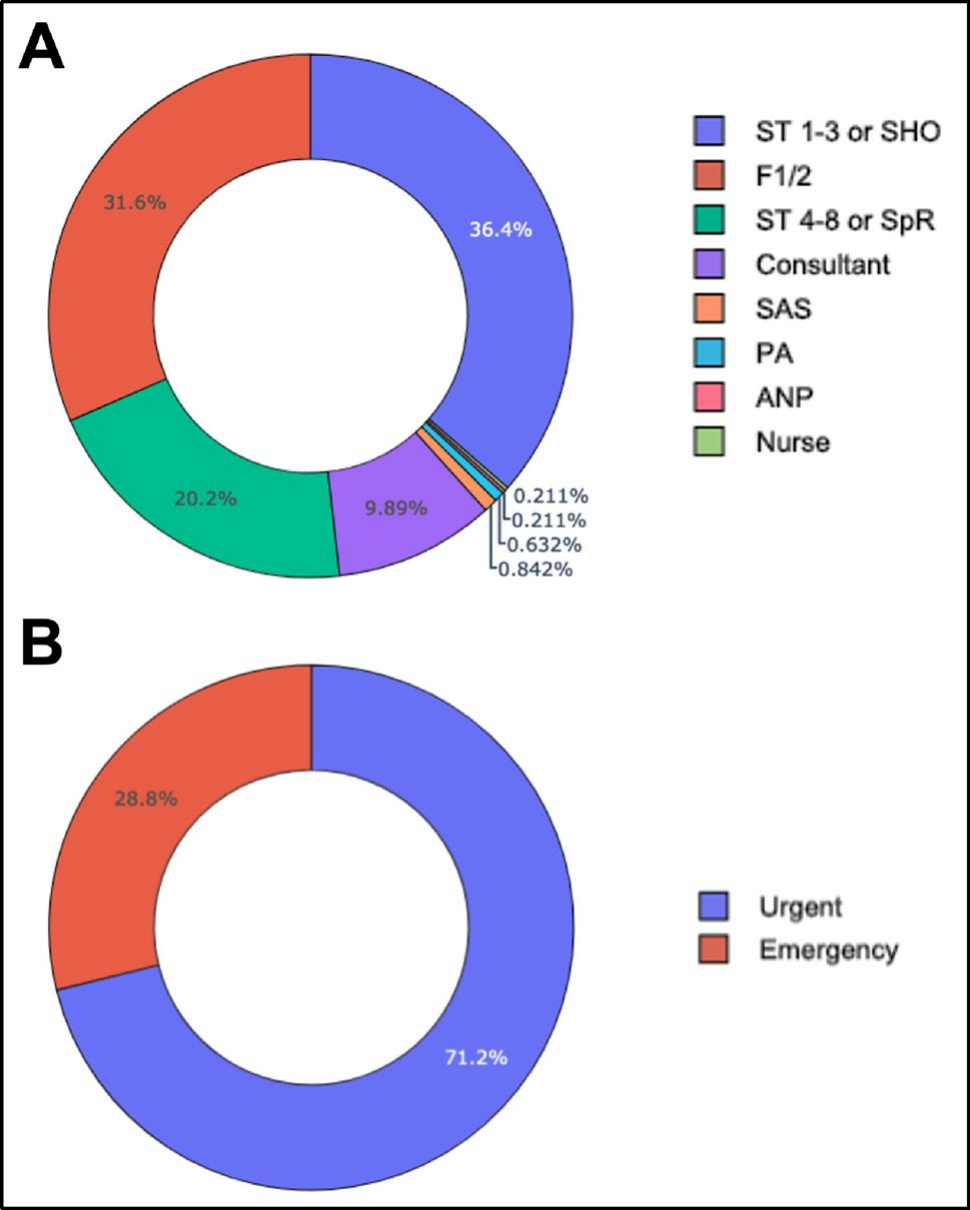
Referral breakdown by **A** grade of referring clinician and **B** urgency. (*ST* specialist trainee, *SHO* senior house officer; *ICU* intensive care unit; *SpR* specialist registrar; *PA* physician associate; *ANP* advanced nurse practitioner)

The majority of referrals were made by acute medical and emergency specialities, in addition to endocrine and neurology teams (Fig. 2). The most common referring grade was a senior house officer (junior resident) or foundation year doctor or intern (Fig. 3A). Approximately 30% of referrals were conveyed by a senior registrar or consultant (attending) and a small handful by allied health specialities, although the decision to refer would in all cases have a consultant’s name attached. 29% of referrals were labelled as an ‘emergency’ by the referring team (Fig. 3B).

#### Breakdown by presentation, lesion origin and radiological findings

**Fig. 4:**
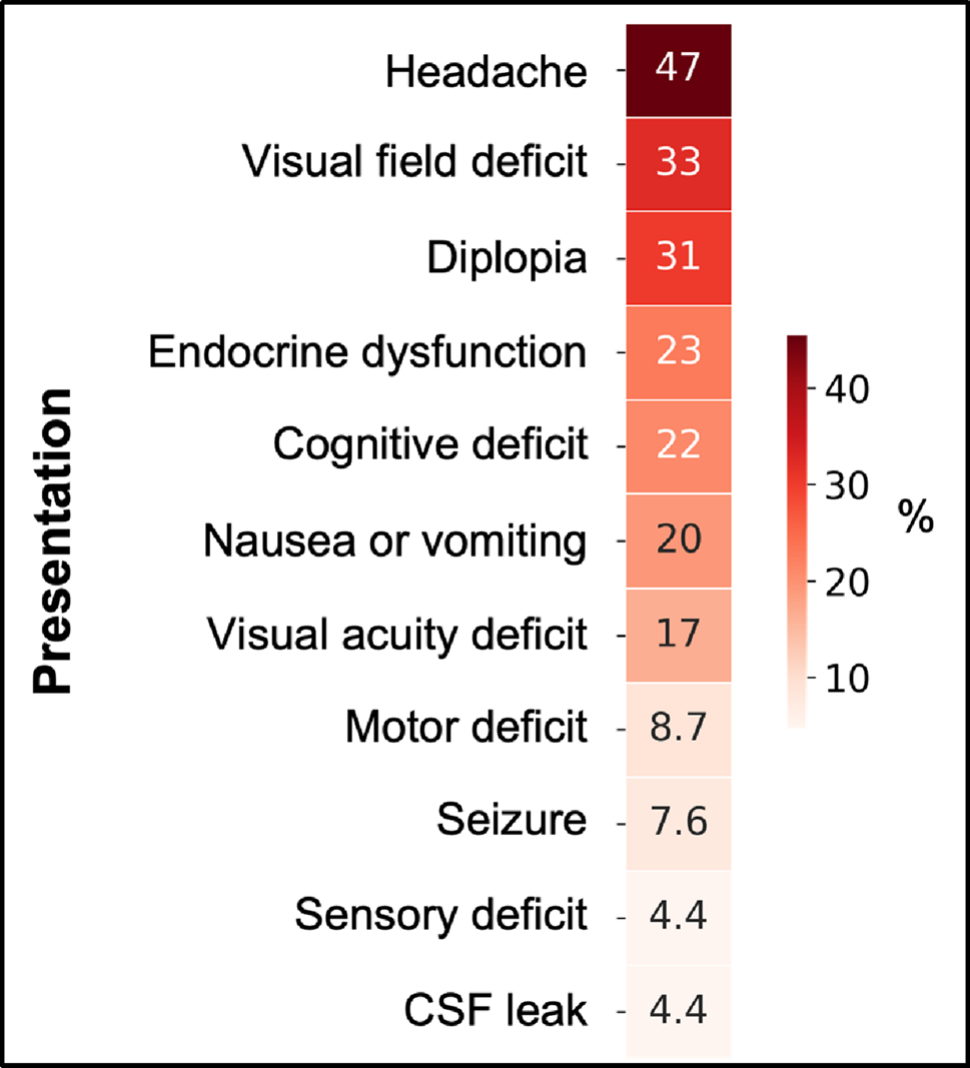
Frequency heat map of presentations for acute pituitary referrals. Numbers in the heatmap correspond to the respective percentage frequency for each symptom. ‘Cognitive deficit’ symptoms referred to confusion, loss of consciousness or disorientation; ‘Endocrine dysfunction’ presentations included referral due to an abnormal hormone or biochemical level or endocrine symptoms such as galactorrhoea; ‘CSF leak’ included any rhinorrhea or salty post-nasal drip with a high index of suspicion for being CSF—such as a post-operative or traumatic history, positive imaging or β2-transferrin result

**Fig. 5:**
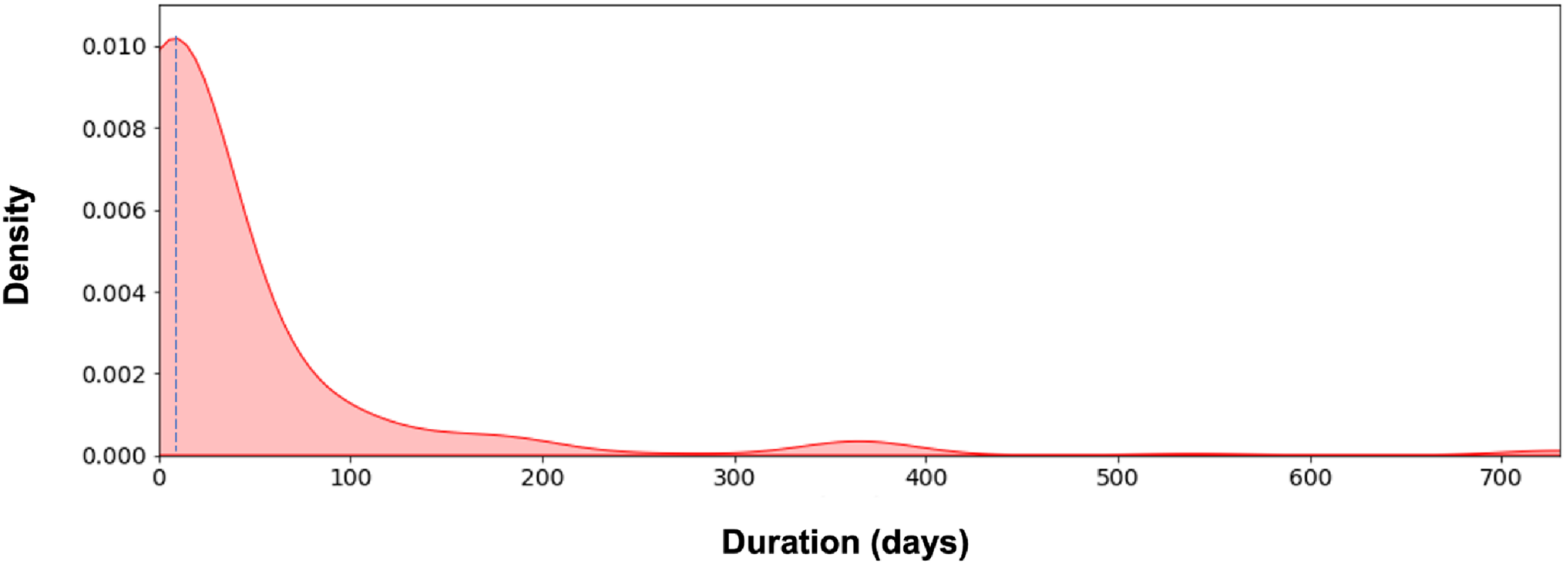
Density distribution of symptom duration prior to referral. Median symptom duration shown as the blue dashed line

26% of patients were symptomatic (n = 112). The most common referral presentations were headache and visual field deficits such as hemianopia (Fig. 4). 4.1% of referrals were incidentally identified on neuroimaging on investigation for another complaint. In 6% of patients, pituitary apoplexy was suspected by the referring team, either based on imaging alone or in conjunction with clinical presentation. The median duration of symptoms prior to referral was 6.5 days (range = 1–730) [Fig. 5], although this varied widely depending on the pattern of symptoms.

92% of referrals had attached some radiological information (transferred scan and/or report) either at the point of, or on completion of the referral. This included CT (62.2%) or MRI (50.0%), although in many cases more than one modality of imaging information was available. A small but non-trivial number had completed angiographic or radioisotope imaging, typically as part of the investigation for another complaint. This included a CT angiogram (2.3%), CT venogram (1.5%), MR venogram (1.7%) and PET scans (1.3%).

**Fig. 6:**
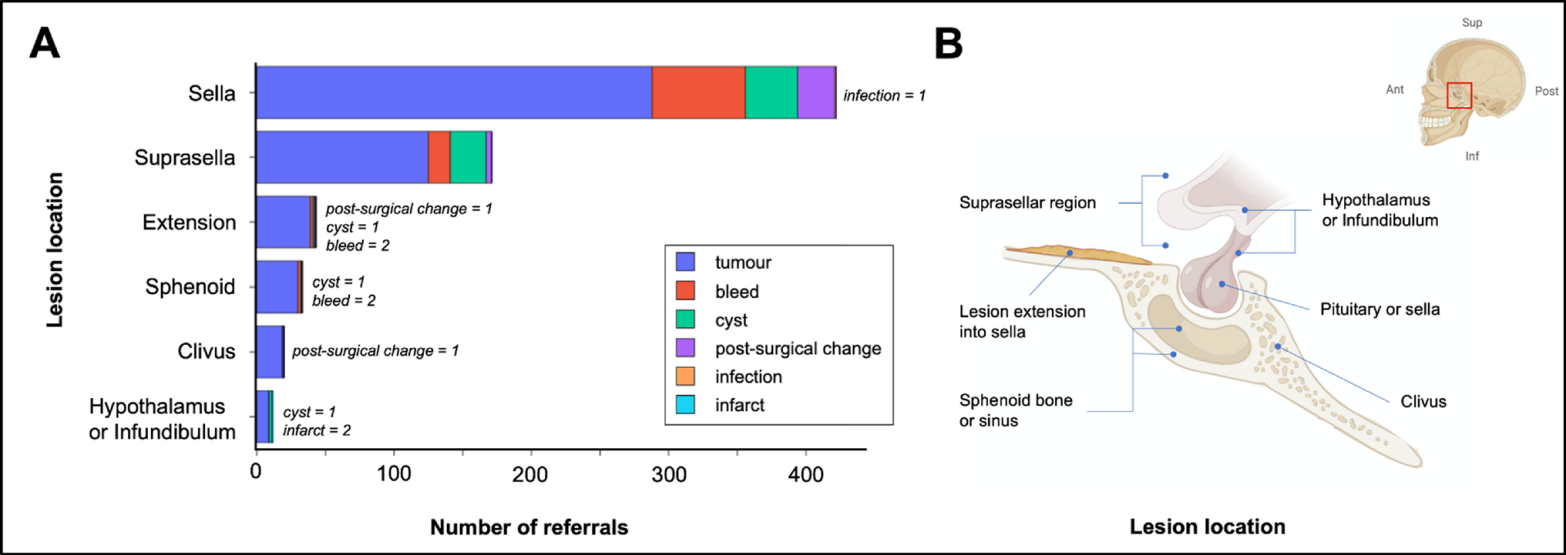
Referrals classified by anatomy and radiological description (**A**) with corresponding lesion locations (**B**). (‘Tumour’ includes radiological descriptors of adenoma, mass, tumour. ‘Cyst’ referred to any lesion which was partially or entirely cystic. ‘Post-surgical’ describes postoperative changes seen on imaging. ‘Extension’ described a lesion which appeared to originate from outside of the other regions e.g. planum sphenoidale or cavernous sinus, and was found to track into the sella or suprasellar space. Note that each patient may have more than one type of radiological descriptor. *Ant* anterior; *Sup* superior; *Post* posterior; *Inf* inferior. Image produced using BioRender)

Lesions mainly comprised tumours (n = 325, 70%), haemorrhage (n = 20, 4%) or both (n = 49, 11%) (Fig. 6). Other lesion types included cysts (n = 52, 11%), with a handful of patients with a suspected infection or infarction. Anatomically, lesions frequently affected the pituitary gland or pituitary fossa (n = 324, 70.1%), suprasellar space (n = 133, 28.8%) and extended into the sella from an extra-axial origin (n = 42, 9.0%), with several patients having lesions which involved multiple anatomical regions on presentation.

#### Model performance

**Fig. 7:**
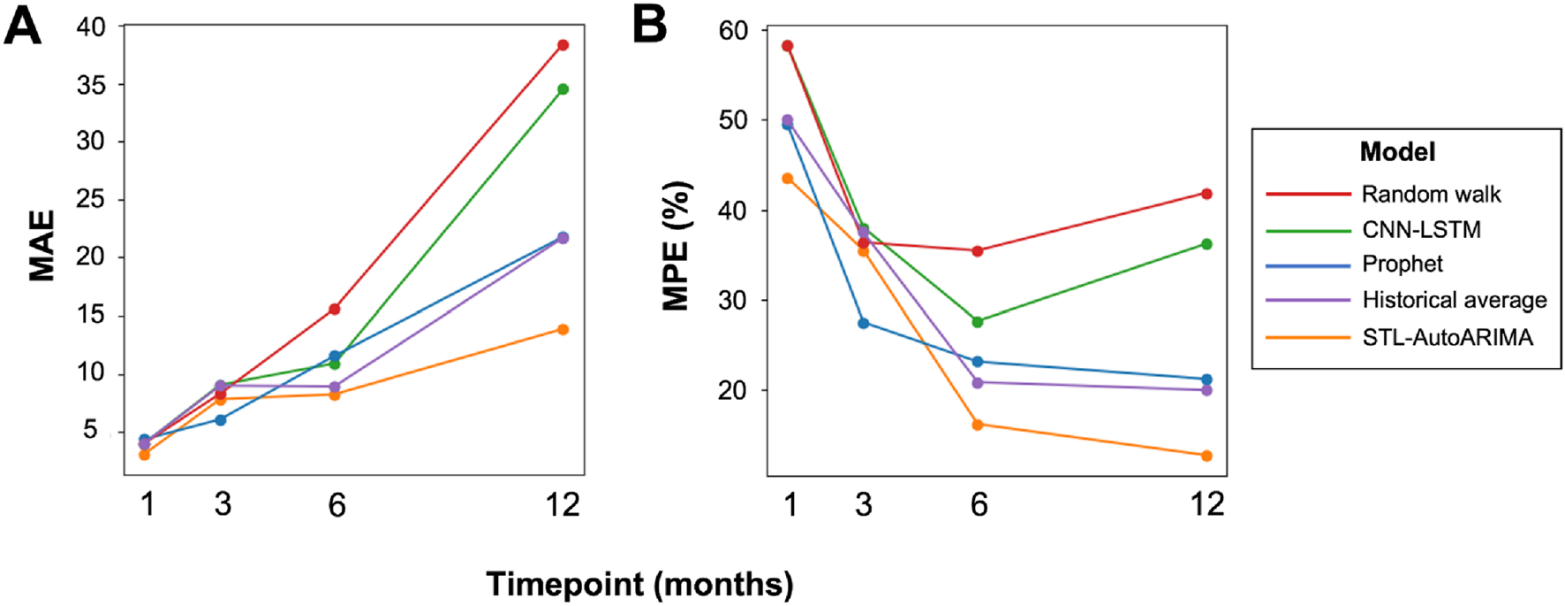
Time-series model performance using median absolute errors (**A**) and median absolute percentage errors (**B**). *CNN* convolutional neural network; *LSTM* long short-term memory; *STL* Seasonal and Trend decomposition using Loess; *ARIMA* auto-regressive integrated moving average

On both median absolute and median absolute percentage error metrics for all timeframes, the STL-AutoARIMA pipeline outperformed the neural network and Prophet algorithms in forecasting acute pituitary referrals (Fig. 7; Supplementary Table 1). As might be anticipated, the median percentage error was lowest for longer time-frames, but only the STL-AutoARIMA and Prophet algorithms reached an acceptable range of accuracy (< 25% MAPE) after 6 months [16]. When compared to the baseline forecasting methods, STL-AutoARIMA significantly outperformed both the random walk and historical average across almost all time frames (Supplementary Table 2).

#### Referral time-series and forecasting

**Fig. 8:**
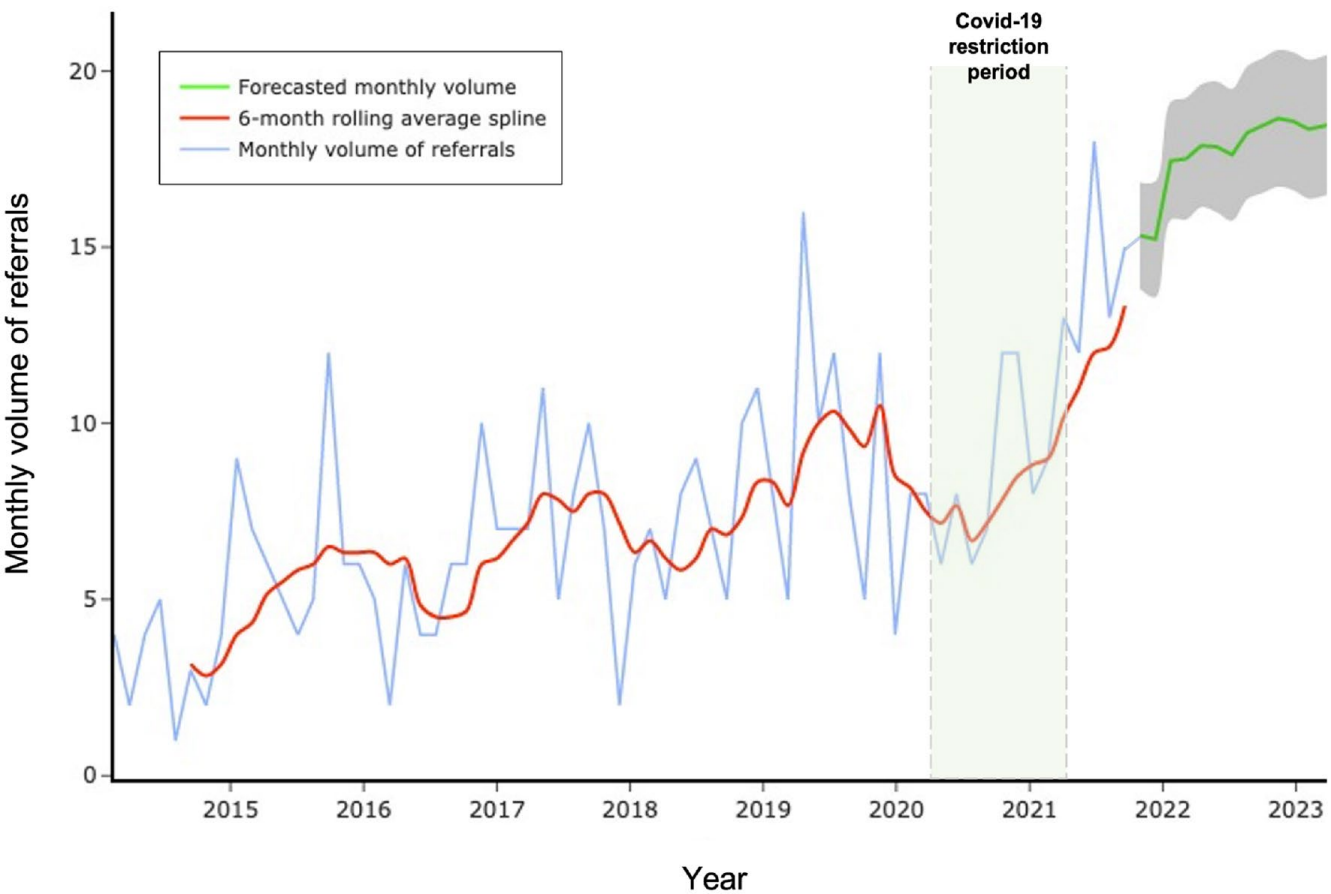
Time-series and forecasted emergency pituitary referrals. The period of Covid-19 governmental lockdown restrictions is highlighted in pale green between March 2020 and February 2021. STL-ARIMA forecasted referrals are shown in dark green with confidence intervals on this prediction in grey

**Fig. 9:**
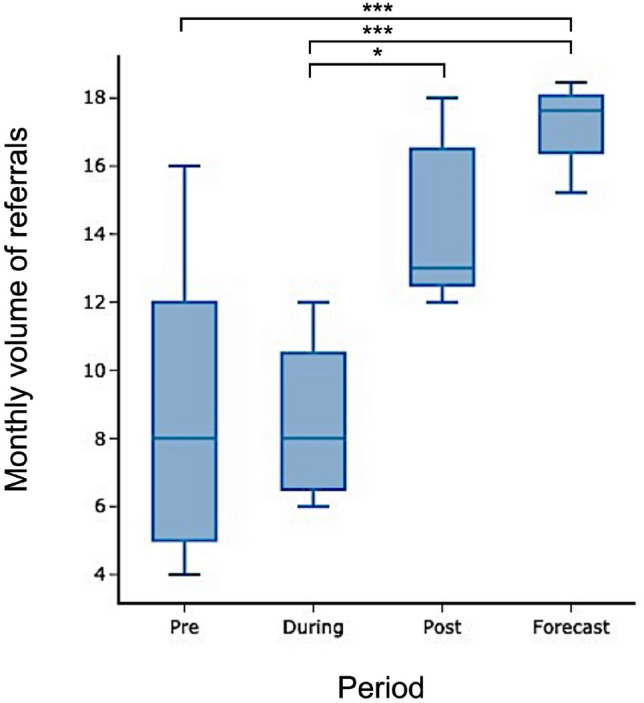
Boxplots of median referral volume for the pre-, during and post-Covid-19 restricted, and 12 month forecasted periods. (*p < 0.05; ***p < 0.001, following post-hoc Dunn test and multiple comparison correction)

Median monthly referral volumes increased in the period ranging from before the Covid-19 pandemic through to a forecasted period approximately 18 months after governmental restrictions ceased (Kruskal Wallis 21.2, p < 0.0005) (Figs. 8 and 9). Post-hoc differences (Dunn’s test with multiple comparison correction) were found between: Covid-19 and post-Covid-19 (p < 0.05); pre-Covid-19 and forecasted, and between Covid-19 and forecasted periods (both p < 0.0005) (Fig. 9). There were no significant differences between pre- and Covid-19 periods, suggesting that the volume of acute pituitary referrals was relatively unaffected during this time.

Unique referring hospitals did not vary across these time periods (KW 4.0, p = 0.13) nor did GCS of the referred patient (KW 3.6, p = 0.17) or the proportion of emergency to urgent patients (χ^2^ 2.7, p = 0.26). While symptom duration significantly varied across time periods (KW 7.6, p = 0.02), pairwise differences did not meet the threshold for significance. In contrast, when referring doctors were aggregated into interns (FY), residents (SHO, SpR) and attendings, there were significant proportional differences with time (χ^2^ 11.9, p = 0.01). This was found to be driven by differences between the pre and during Covid-19 periods (χ^2^ 9.7, p = 0.02) and between pre- and post-Covid-19 periods (χ^2^ 6.0, p = 0.07) following multiple comparison correction. Likely underpinning these differences was the drop in residents conveying referrals during and after the pandemic coupled with a smaller increase in interns (Table 2).

**Table 2:** Referral phenotype in the 12-month period before and during the Covid-19 pandemic, the period in which lockdown governmental restrictions ceased for which data was available (Post Covid -19) and in the forecasted 12 months after this

	Pre Covid-19	Covid-19	Post Covid-19	12 month forecast
Time interval	March 2019–Feb 2020	March 2020–Feb 2021	March 2021–September 2021	October 2021–September 2022
Median monthly referral volume (IQR)	8 (5–12)	8 (7–9)	13 (12–15)	18 (17–18)
Median unique monthly referring sites (IQR)	5 (4.8–6.3)	4 (2.8–5.3)	5 (5–7.5)	–
Mean glasgow coma score (SD)	14.8 (0.6)	14.7 (1.6)	14.8 (0.7)	–
Urgent: emergency (%)	70:30	65:35	78:22	–
Median symptom duration (IQR)	3 (1–14)	7 (1–28)	9.5 (3–40.5)	–
Referrer grade (Intern/Resident/Attending)	23/50/8	30/22/10	31/28/6	–

## Discussion

### Principal findings

Firstly, this study provides a detailed descriptive analysis of contemporary electronic referral data from a large pituitary referral centre. This has allowed for the audit of a complex referral pathway, which includes mapping of the geographical catchment, presentation characteristics, and referral attributes such as the referring professional’s grade and accompanying investigations. The majority of referrals came from relatively local centres and were conveyed by junior referring doctors. Future growth of the service, likely related to the consolidation of pituitary services and increased availability of imaging, has been forecasted and validated to continue for the next year. This research highlights several areas for quality improvement and further evaluation in our referral network, including: (i) increasing the proportion of referrals with relevant imaging; (ii) helping focus district educational initiatives on certain areas; and (iii) planning service adaptations to forecasted trends.

Secondly, the influence of the COVID-19 pandemic on referral trends was explored. Surprisingly, the reduction in referral volume during the COVID-19 pandemic did not reach statistical significance. This was accompanied by significant workforce changes, with less residents making referrals when compared to other grades, likely reflecting deployment to COVID care services. The consistent referral volume despite this is in contrast to neurosurgical referrals for other acute cranial and spinal pathologies, which experienced a considerable reduction in referrals [17–19]. The frequent sight or life-threatening presentation of pituitary tumours and anatomically-adjacent pathologies in this cohort could explain this [1, 6] and may be a sufficient reason for patients to present, overcoming various fears associated with hospitals and COVID-19 transmission during the pandemic [20, 21]. Nevertheless, the number of pituitary and skull base surgeries performed in the UK did decrease during the COVID pandemic, including in our centre. This occurred in the context of limited operating capacity, an evolving understanding of the theorised viral transmission risk of these surgeries, and the development of updated clinical guidelines for the management of these tumours [6, 8, 22], all impacting upon surgical decision-making and technique. Consequently, a significant proportion of patients with pituitary disease were affected, resulting in delays or changes to their planned care [8, 22]. Furthermore, the steep rise in referrals post-pandemic may reflect this backlog, with patients presenting in a delayed fashion as COVID-related fears and barriers to healthcare access decrease. The current study characterises the pandemic impact on pituitary and skull base referrals, which may aid prediction of, and preparation for, future stressors to the service [17].

Finally, machine-learning driven forecasting of anticipated referral volume will be useful in preparing surgical services for the future. Here, our STL-AutoARIMA model outperformed both the CNN and Prophet models across all time-periods. Field-standard accuracy levels were achieved for 6-month (and longer) forecasts and accounted for time-series volatility associated with the COVID-19 pandemic [16]. We found an increase in the volume of referrals after the pandemic, and an increasing trend throughout, likely secondary to greater detection of lesions through increasingly available magnetic resonance imaging, coupled with systems factors such as consolidation of services. These time-series predictions can directly feed into planning service expansion to meet increases in surgical demand and enable mapping of the skull base rota cover to referral trends in the short-term. The automated nature of these analyses will allow this service planning to be efficient, dynamic and up-to-date and would help contribute to a widely-held objective of a flexible, dynamic surgical department [23]. This study highlights the potential of ML in pituitary disease, complementing previous applications including the prediction of recurrence in Cushing’s disease or image-analysis of pituitary tumours [24, 25].

### Findings in the context of existing literature

Large-scale population studies remain fundamental in understanding epidemiology, treatment patterns, and the burden of pituitary disorders, which ultimately inform service needs [26, 27]. Evolving international data continues to support the consolidation of pituitary and skull base services into regional referral centres with the necessary multidisciplinary infrastructure and surgical volume [2, 7, 28–30]. This builds on the “centres of excellence” model proposed by the Pituitary Society, which recommends dedicated fellowships and high-volume practice for pituitary neurosurgeons [2, 3]. All in all, these changes will increase referral and case volume at each dedicated centre, and as centres’ services continue to consolidate, patient pathways and service needs will be dynamic and increasingly complex [7]. Data-driven review of practice and accurate analysis of trends will therefore be crucial to meet these demands [7].

The first step in this process is the use of an electronic referral system, which improves communication efficiency, referral transparency, and access; data entry and integration with analysis software [31]. However, the input of complete and relevant data to each referral is still a challenge, regardless of whether a system is electronic or paper based. Other studies of neurosurgical referral networks have also recognised the attachment or completion of relevant imaging studies as a recurring issue [32]. Clear clinical guidelines for referrers and referrer education programmes are among the proposed mitigation strategies [33, 34]. Our centre has implemented an annual event within our referral network directed at emergency department physicians, with an audit of the impact on referral quality ongoing [33, 34].

There are several examples in the surgical literature of time-series forecasting being used [35, 36], but few have trained on acute surgical service or referral data [37]. More generally across healthcare, others have used the ARIMA model alone [38] or in combination with neural networks [39] to make time-series predictions. Our approach using a STL-ARIMA pipeline is initially compared against novel candidates (both neural network and additive algorithms). Other than the manual calibration step of the COVID-19 lockdown period, it is fully automated in both decomposition and hyperparameter tuning. This allows the model to dynamically adjust to the data volume and seasonality. Several steps were taken in the study to reduce overfitting including dropout layers and block cross-validation [40]. However, further confirmation of the model’s generalisability would require sufficient data in order to perform a train-test split and other data sources for external validation.

### Strengths and limitations

This study encompasses referral data from the largest pituitary centre in the UK, with a large and diverse network population. It provides a comprehensive assessment of emergency and urgent pituitary referrals and is the first study to forecast pituitary referrals and guide providers in service planning. Nevertheless, we acknowledge several limitations. Firstly, the study is single-centre, and future multicentre work (including studies outside of the UK) will be needed to improve the external validity of our findings [28]. Secondly, the electronic referral data requires substantial preprocessing and qualitative synthesis prior to analysis, with some referrals missing relevant data (e.g., attached imaging). Thus, an improvement in data proforma design and data entry compliance will facilitate more seamless future analyses. Thirdly, because the electronic referral data were unmatched, outcomes of interest including the proportion of patients who underwent surgery and their respective histopathological diagnoses, outcomes, complications and other salient demographic information such as ethnicity were unavailable, which would be useful when streamlining service pathways. Finally, our forecasting model only achieved ‘standard’ accuracy at 6 months or more and failed to significantly outperform one standard baseline model. This limits the ability of the pipeline to offer insights, particularly in the short-term and facilitate rapid service changes in resource allocation. Further, iterative improvements would require larger datasets, over longer time periods, and wider hyperparameter optimisation, including the trial of different optimisation methods or the use of ensemble techniques [35]. Adding more information into the forecasting model such as acuity of the referral and relevant clinical or radiological information would help gain greater precision into the type of referral received. We note that patient age in particular had a large standard deviation, which suggests there may be differences in referrals based on the demographics of the patient.

## Conclusions

As pituitary and skull base centres continue to merge, referral volume and network complexity will increase. These dynamic networks can be accurately analysed using electronic referral databases and machine-learning models. Using these techniques, an accurate data-driven audit of current referral characteristics and forecasting of future service demand was performed at the UK’s largest pituitary centre. Iterative enhancements to patient databases and machine learning models will be made in the future, as will multicentre studies of longer-term data. This technology could help in bridging the gap to more digitally-augmented health care systems, and could be applied across pathologies, disciplines, and centres in the future.

## Supplementary Information

Below is the link to the electronic supplementary material.

Supplementary file1 (DOCX 473 KB)

## Data Availability

Patient data is available on reasonable request to the corresponding author, providing appropriate ethical and data governance permissions have been obtained.

## References

[1] Pal A, Leaver L, Wass J (2019). Pituitary adenomas. BMJ.

[2] Casanueva FF, Barkan AL, Buchfelder M (2017). Criteria for the definition of Pituitary Tumor Centers Of Excellence (PTCOE): a pituitary society statement. Pituitary.

[3] McLaughlin N, Laws ER, Oyesiku NM (2012). Pituitary centers of excellence. Neurosurgery.

[4] Surchi H, Jafar-Mohammadi B, Pal A (2017). Local optometrists are a major source of referrals to a pituitary tumour clinic. Endocr-Relat Cancer.

[5] Petrossians P, Daly AF, Natchev E (2017). Acromegaly at diagnosis in 3173 patients from the Liège Acromegaly Survey (LAS) Database. Endocr-Relat Cancer.

[6] Varlamov EV, Niculescu DA, Banskota S (2021). Clinical features and complications of acromegaly at diagnosis are not all the same: data from two large referral centers. Endocr Connect.

[7] Mortini P, Nocera G, Roncelli F (2020). The optimal numerosity of the referral population of pituitary tumors centers of excellence (PTCOE): a surgical perspective. Rev Endocr Metabolic Disord.

[8] Khan DZ, Marcus HJ, Consortium C (2021). CSF rhinorrhoea after endonasal intervention to the skull base (CRANIAL)—part 1: multicenter pilot study. World Neurosurg.

[9] Pandit AS, Jalal AHB, Toma AK, Nachev P (2022). Analyzing historical and future acute neurosurgical demand using an AI-enabled predictive dashboard. Sci Rep-UK.

[10] Collins GS, Reitsma JB, Altman DG, Moons KGM (2015). Transparent reporting of a multivariable prediction model for individual prognosis or diagnosis (TRIPOD): the TRIPOD statement. Bmj Br Medical J.

[11] Box GEP, Jenkins GM, Reinsel GC, Ljung GM (2016). Time series analysis forecasting and control.

[12] Hochreiter S, Schmidhuber J (1997). Long short-term memory. Neural Comput.

[13] Sainath TN, Vinyals O, Senior A, Sak H (2015) Convolutional long short-term memory, fully connected deep neural networks. In: 2015 IEEE international conference on acoustics, speech and signal processing (ICASSP) 4580–4584. 10.1109/icassp.2015.7178838

[14] Taylor SJ, Letham B (2018). Forecasting at scale. Am Stat.

[15] Hyndman R, Athanasopoulos G (2018). Forecasting: principles and practice.

[16] Swanson D (2015). On the relationship among values of the same summary measure of error when it is used across multiple characteristics at the same point in time: an examination of MALPE and MAPE. Rev Econ & Finance.

[17] Pandit A, Jalal A, Toma A, Nachev P (2022) An AI-enabled predictive analytics dashboard for acute neurosurgical referrals. 10.21203/rs.3.rs-1216653/v1 10.1038/s41598-022-11607-9 PMC9084272 35534601

[18] Horan J, Duddy JC, Gilmartin B (2021). The impact of COVID-19 on trauma referrals to a National Neurosurgical Centre. Ir J Medical Sci.

[19] Sinha S, Toe KKZ, Wood E, George KJ (2021). The impact of COVID-19 on neurosurgical head trauma referrals and admission at a tertiary neurosurgical centre. J Clin Neurosci.

[20] Fleseriu M, Dekkers OM, Karavitaki N (2020). Endocrinology in the time of COVID-19: management of pituitary tumours. Eur J Endocrinol.

[21] Graf A, Marcus HJ, Baldeweg SE (2021). The direct and indirect impact of the COVID-19 pandemic on the care of patients with pituitary disease: a cross sectional study. Pituitary.

[22] Bandyopadhyay S, Khan DZ, Marcus HJ (2021). CSF rhinorrhea after endonasal intervention to the skull base (CRANIAL)—part 2: impact of COVID-19. World Neurosurg.

[23] Kerr RS (2020). Surgery in the 2020s: implications of advancing technology for patients and the workforce. Futur Heal J.

[24] Saha A, Tso S, Rabski J (2020). Machine learning applications in imaging analysis for patients with pituitary tumors: a review of the current literature and future directions. Pituitary.

[25] Nadezhdina EY, Rebrova OYu, Grigoriev AY (2019). Prediction of recurrence and remission within 3 years in patients with Cushing disease after successful transnasal adenomectomy. Pituitary.

[26] Burton T, Nestour EL, Neary M, Ludlam WH (2016). Incidence and prevalence of acromegaly in a large US health plan database. Pituitary.

[27] Broder MS, Neary MP, Chang E (2015). Treatment patterns in Cushing’s disease patients in two large United States nationwide databases: application of a novel, graphical methodology. Pituitary.

[28] Phillips N (2018) Cranial Neurosurgery—GIRFT Programme National Specialty Report

[29] Wass J, Lansdown M (2021) Endocrinology—GIRFT Programme National Specialty Report

[30] SBNS (2021) Neurosurgical National Audit Programme. Available at https://www.nnap.org.uk. Accessed on 31 Mar 2022

[31] Amarouche M, Neville JJ, Deacon S (2017). Referrers’ point of view on the referral process to neurosurgery and opinions on neurosurgeons: a large-scale regional survey in the UK. BMJ Open.

[32] Fountain DM, Davies SCL, Woodfield J (2019). Evaluation of nationwide referral pathways, investigation and treatment of suspected cauda equina syndrome in the United Kingdom. Br J Neurosurg.

[33] Pradini-Santos L, Craven CL, Usher I (2020). A novel neurosurgery referral course: feasibility, validation, and inferences for patient care. J Surg Educ.

[34] Haneef Z, Stern J, Dewar S, Engel J (2010). Referral pattern for epilepsy surgery after evidence-based recommendations. Neurology.

[35] Kaushik S, Choudhury A, Sheron PK (2020). AI in healthcare: time-series forecasting using statistical, neural, and ensemble architectures. Front Big Data.

[36] Wang J, Vahid S, Eberg M (2020). Clearing the surgical backlog caused by COVID-19 in Ontario: a time series modelling study. CMAJ.

[37] Chandrabalan V, Sim N, Peristerakis I, Beveridge AJ (2021). The application of time-series forecasting to quantify the deficit in colorectal 2-week wait referrals caused by the COVID19 pandemic. Colorectal Dis.

[38] Rodea-Montero ER, Guardado-Mendoza R, Rodríguez-Alcántar BJ (2021). Trends, structural changes, and assessment of time series models for forecasting hospital discharge due to death at a Mexican tertiary care hospital. PLoS ONE.

[39] Zhai M, Li W, Tie P (2021). Research on the predictive effect of a combined model of ARIMA and neural networks on human brucellosis in Shanxi Province, China: a time series predictive analysis. Bmc Infect Dis.

[40] Stevens LM, Mortazavi BJ, Deo RC (2020). Recommendations for reporting machine learning analyses in clinical research. Circ Cardiovasc Qual Outcomes.

