# Supplementary material for: Historical and future trends in emergency pituitary referrals: a machine learning analysis
Source: Pituitary. 2022 Sep 9;25(6):927–37. doi: 10.1007/s11102-022-01269-1 (PMC9462621; doi:10.1007/s11102-022-01269-1)
Supplement: Supplementary file 1 — Supplementary file1 (DOCX 473 KB) [file 11102_2022_1269_MOESM1_ESM.docx]

Historical and Future Trends in Emergency Pituitary Referrals: a Machine Learning Analysis

# Supplementary Material

## Exploratory data analysis

Given the wide standard deviation within the referring patients’ ages, their distribution was further explored (Supplementary Figure 1). The distribution was tested using Hartigan’s dip test to find that the data was not significantly bimodal (dip statistic = 0.02, p = 0.68). As such further discretized analysis (e.g. young vs. old patients) was not performed in this study.

**Supplementary Figure 1.** Combined kernel density estimation and histogram of patient ages.


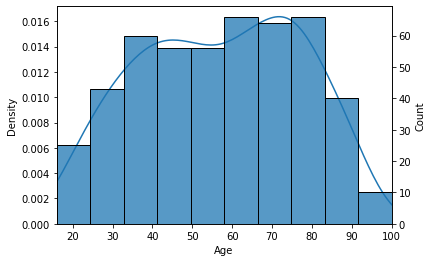


## Time-series forecasting

The time-series methods in the main manuscript are discussed here in greater detail, and code is also included.

### STL + AutoARIMA

We performed an exploratory analysis of the time-series using auto-correlation and partial auto-correlation plots in combination with augmented Dickey-Fuller testing to determine the degree of stationarity in the data and assist in defining initial parameters for seasonal decomposition and upper and lower parameter limits for the auto-ARIMA grid search.

<CODE>

| *### STL/Auto-ARIMA model* *#Run EDA on monthly time-series first to manually check seasonality*  *#Set variables* res = []  *#STL period corresponds to expected seasonality. 12 chosen to reflect monthly seasonal changes.* period = 12  *#How long into future/out-of-sample to make forecast* future = 0 *#95% Confidence interval* confidence = 0.05  *#STL decomposition with default parameters and period - can be further tuned using grid search* res = STL(df, period = period, robust = False).fit()  *#Seasonal auto-ARIMA can be changed to True for more thorough grid search. Upper and lower limits regarding p, q, d determined by initial exploratory analysis of data set* smodel = pm.auto_arima(res.seasonal,  start_p=0, max_p=5,  start_q=0, max_q=5,  seasonal=False,  stepwise = False,  start_d=0, max_d=5,  trace=False, error_action='ignore');  *#Trend auto-ARIMA* tmodel = pm.auto_arima(res.trend,  start_p=0, max_p=5,  start_q=0, max_q=5,  seasonal=False,  stepwise = False,  start_d=0, max_d=5,  trace=False, error_action='ignore');  *#Residual auto-ARIMA* rmodel = pm.auto_arima(res.resid,  start_p=0, max_p=5,  start_q=0, max_q=5,  seasonal=False,  stepwise = False,  start_d=0, max_d=5,  trace=False, error_action='ignore');  *#Modelling seasonality* modelsea = SARIMAX(res.seasonal, order = smodel.order, seasonal_order= smodel.seasonal_order).fit()  *#If Auto-ARIMA fails then use simple differenced d=1 model for trend and residual components* try:  modeltrend = ARIMA(res.trend, order = tmodel.order, freq=interval).fit() except:  modeltrend = ARIMA(res.trend, order = (0,1,0), freq=interval).fit()   *#Modelling residual* try:  modelres = ARIMA(result.resid, order = rmodel.order).fit() except:  modelres = ARIMA(result.resid, order = (0,1,0)).fit()  *#Forecasting*  forecast_season = modelsea.forecast(future, alpha=confidence) forecast_trend, std_err_trend, confidence_int_trend = modeltrend.forecast(future, alpha=confidence) forecast_resid, std_err_resid, confidence_int_resid = modelres.forecast(future, alpha=confidence)  *#recomposition* forecast_final = forecast_season + forecast_trend + forecast_resid conf = confidence_int_trend + confidence_int_resid |
| --- |

### CNN - LSTM

<CODE>

| *###CNN-LSTM implementation*  *#Relevant imports* from tensorflow.keras.models import Sequential from tensorflow.keras.layers import LSTM, Dense, Flatten, TimeDistributed, Conv1D, MaxPooling1D  *# define input sequence from dataframe* sequence = df['all'].to_list()  *# Set number of steps, keep even* n_steps = 12  *# split into an array of subsequences, X = input* X, y = sequence_split(sequence, n_steps)  features = 1 n_seq = 2  *# divided subsequence into 2 subsamples* n_steps2 = n_steps/2  *# reshape input data for CNN layer* X = X.reshape((X.shape[0], n_seq, n_steps2, features))  *# set up sequential stack model* model = Sequential()  *#CNN layer with 64 output filters, kernel size corresponds to length of convolutional window. Input shape must match shape from reshape step* model.add(TimeDistributed(Conv1D(filters=64, kernel_size=1, activation='relu'), input_shape=(None, n_steps2, n_features)))  *# Down samples by pool size* model.add(TimeDistributed(MaxPooling1D(pool_size=2)))  *#Flatten to single 1D vector* model.add(TimeDistributed(Flatten()))  *#Single LSTM layer with 64 neurons* model.add(LSTM(64, activation='relu'))  *#NN dense layer* model.add(Dense(1))  *#ADAM optimisation using mse as a cost function. Default parameters / adaptive learning rate chosen to facilitate generalisatbility of the model*  model.compile(optimizer='adam', loss='mse') model.fit(X, y, epochs=500, verbose=0)  *## RELEVANT PROCESSING FUNCTIONS*  def sequence_split(timeseries, n_steps):    *#Prepare list variables*  X, y = list(), list()    for i in range(len(timeseries)):    *# find index at sequence end*  end_index = i + n_steps    *# stop code if has gone past total length of sequence*  if end_index > len(timeseries)-1:  break    *# divide sequence into subsamples*  sub_x, sub_y = timeseries[i:end_index], timeseries[end_index]  X.append(sub_x)  y.append(sub_y)    return np.array(X), np.array(y) |
| --- |

Loss curves were generated for the CNN-LSTM model to ensure an adequate learning rate and number of epochs using the optimiser hyperparameters described in the code (see Supplementary Figure 2).

**Supplementary Figure 2.** CNN-LSTM Loss curves when training on the full data set (orange) and mean averaged cross-validated sub-datasets (blue)


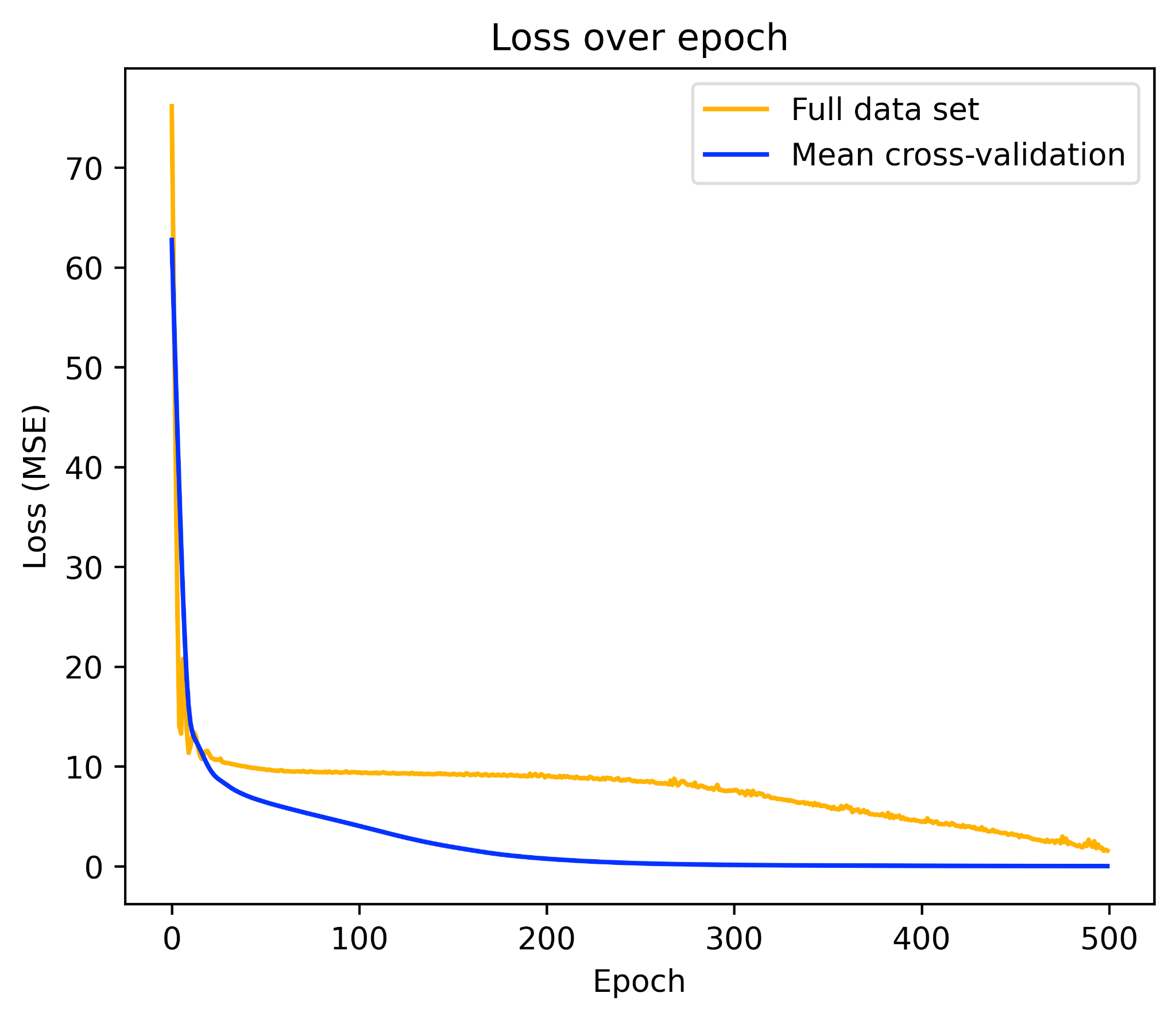


##

### Prophet

<CODE>

| *### Prophet implementation and imports*  from prophet import Prophet  *#Specify dataframe and convert to prophet input*  prophetdf = df.reset_index() prophetdf.columns = ['ds', 'y']  *#Specify out-of-sample months to predict* prediction = 1  *#Specify COVID-19 lockdown period - code block can be toggled*  lockdown = pd.DataFrame({  'holiday': 'lockdown',  'ds': pd.to_datetime(['2020-03-23']),  'lower_window': 0,  'upper_window': 84,  })  *#Set model parameters. Note weekly seasonality*  model = Prophet(yearly_seasonality=True,  weekly_seasonality=True,  seasonality_mode='additive',  interval_width=0.95,  changepoint_prior_scale= 0.05,  seasonality_prior_scale= 0.1,  holidays = lockdown)  *#Fit model* model.fit(prophetdf) future = model.make_future_dataframe(periods=prediction,freq='M')  *#Make predictions* forecast = model.predict(future) |
| --- |

## Comparison against baseline models

| def random_walk(start, length, stdev, tsmin, tsmax):    rw = [start]  for t in range(1,length):  *#random step is constrained at an upper limit by standard deviation of the dataframe. Note ‘rando’ can be positive or negative.*  rando = np.random.normal(0,stdev)  yhat = rw[t-1] + rando  *#Section below can be toggled to further constrain the random walk to the lower and upper limits of the sub-dataset*  if yhat > tsmax:  yhat = rw[t-1] - abs(rando)  elif yhat < tsmin:  yhat = rw[t-1] + abs(rando)  rw.append(yhat)   return rw |
| --- |
|  |

## Exploratory data analysis

**Supplementary Table 2.** **Cross validation error metrics for each algorithm, calculated for various lengths of forecast.** (MAE = median absolute error; MAPE = medIan absolute percentage error; STL = seasonal trend Loess decomposition; ARIMA = auto-regressive integrated moving average; CNN = convolutional neural network; LSTM = long short-term memory)

|  |  | **MAE (IQR)** | | | | **MAPE (IQR)** | | | |
| --- | --- | --- | --- | --- | --- | --- | --- | --- | --- |
| **Algorithm** | | 1 | 3 | 6 | 12 months | 1 | 3 | 6 | 12 months |
| Experimental | STL + AutoARIMA | 3.08 (1.29-6.76) | 7.82 (4.25 - 8.70) | 8.24 (5.60 - 16.25) | 13.88 (8.75 - 33.31) | 43.59 (22.03 - 56.35) | 35.50 (20.74 - 39.45) | 16.22 (15.13 - 42.53) | 20.00 (15.33 - 41.75) |
|  | CNN-LSTM | 4.00 (2.00 - 7.00) | 9.06 (3.06 - 10.84) | 10.9 (8.30 - 21.32) | 34.52 (21.18 - 66.62) | 58.33 (33.33 - 60.00) | 38.03 (13.33 - 52.81) | 27.66 (17.91 - 45.01) | 36.21 (27.27 - 61.01) |
|  | Prophet | 4.36 (2.71 - 5.95) | 6.08 (5.84 - 10.95) | 11.56 (1.43 - 20.64) | 21.79 (5.90 - 43.75) | 49.57 (33.89 - 57.40) | 27.53 (25.35 - 51.41) | 23.16 (3.85 - 47.06) | 21.22 (5.87 - 46.55 |
| Baseline | Historical average | 4.00 (2.00 - 7.00) | 9.00 (4.00 - 10.00) | 8.93 (6.75 - 19.00) | 21.71 (13.54 - 40.00) | 50.00 (33.33 - 60.00) | 37.50 (20.00 - 44.00 ) | 20.89 (13.51 - 40.00) | 20.00 (15.33 - 41.75) |
|  | Random walk | 4.00 (2.00 - 7.00) | 8.30 (4.31 - 12.86) | 15.61 (7.05 - 27.36) | 38.42 (17.66 - 66.60) | 58.33 (33.33 - 60.00) | 36.38 (19.81 - 56.19) | 35.47 (17.19 - 61.65) | 41.87 (19.87 - 74.19) |

**Supplementary Table 3.** **Comparison of the STL-AutoARIMA model against baseline forecasting models. (**MAE = median absolute error; MAPE = medIan absolute percentage error. MWU = Mann Whitney U test.)

| **Model comparison** | **Metric** | **Period (months)** | **MWU statistic** | **p** |
| --- | --- | --- | --- | --- |
| Historical average | MAE | 1 | 212360.0 | <0.001 |
|  |  | 3 | 216115.0 | <0.01 |
|  |  | 6 | 227982.0 | 0.2 |
|  |  | 12 | 205843.0 | <0.00001 |
| Random walk | MAE | 1 | 207284.0 | <0.00001 |
|  |  | 3 | 206396.0 | <0.00001 |
|  |  | 6 | 168510.0 | <0.00001 |
|  |  | 12 | 131720.0 | <0.00001 |
| Historical average | MPE | 1 | 204650.0 | <0.00001 |
|  |  | 3 | 210045.0 | <0.0001 |
|  |  | 6 | 225218.0 | 0.144 |
|  |  | 12 | 200927.0 | <0.00001 |
| Random walk | MPE | 1 | 197925.0 | <0.00001 |
|  |  | 3 | 207743.0 | <0.00001 |
|  |  | 6 | 167402.0 | <0.00001 |
|  |  | 12 | 128229.0 | <0.00001 |
